# Lateral Alveolar Ridge Augmentation with Autogenous Tooth Roots and Staged Implant Placement—5-Year Follow-Up Case Series

**DOI:** 10.3390/jcm13175118

**Published:** 2024-08-29

**Authors:** Roko Bjelica, Igor Smojver, Marko Vuletić, Dražena Gerbl, Luka Marković, Dragana Gabrić

**Affiliations:** 1 Private Dental Practice Dr. Marija Stilinović-Bjelica, 10000 Zagreb, Croatia; rbjelica@sfzg.hr; 2 St. Catherine Specialty Hospital, 10000 Zagreb, Croatia; ismojver@gmail.com; 3 Department of Dental Medicine, Clinical Hospital Centre Zagreb, 10000 Zagreb, Croatia; mvuletic@sfzg.hr; 4 Department of Oral Surgery, School of Dental Medicine University of Zagreb, 10000 Zagreb, Croatia; 5 Department of Anaesthesiology and Intensive Care Unit (ICU), University Hospital Centre Zagreb, 10000 Zagreb, Croatia; drazena.gerbl@gmail.com; 6 Marković Dental Clinic, 52100 Pula, Croatia; markovi.luka@gmail.com

**Keywords:** bone regeneration, alveolar ridge augmentation, autografts, dental implants

## Abstract

**Background/Objectives:** Alveolar bone augmentation before implant placement is a safe and effective treatment option for the reconstruction of a deficient alveolar ridge. According to recent research, permanent teeth have been used as bone graft materials, with studies confirming their clinical and histological results. This study aimed to evaluate the efficacy of alveolar ridge augmentation with autogenous tooth roots and staged implant placement, and peri-implant tissue stability in augmented sites. **Methods:** A total of 20 augmentations with autogenous tooth roots on mandibular alveolar ridges in 15 patients were performed. After 6 months, the ridge width (RWa) and ridge width gain (RWg) were measured. Titanium dental implants were placed in grafted sites and loaded 10 weeks after placement. Clinical parameters (bleeding on probing—BOP; probing depth—PD; mucosal recession—MR; and clinical attachment level—CAL) were assessed 2 months (T1), 3 years (T2), and 5 years (T3) after implant loading. **Results:** The mean RWa was 6.71 ± 0.74 mm, and the RWg was 3.15 ± 0.54 mm, respectively. No statistically significant differences were observed for clinical parameters (BOP, PD, MR, and CAL) among different time points (*p* > 0.05). **Conclusions:** Autogenous tooth roots represent a viable solution for alveolar ridge augmentation and implant placement, providing a stable environment for peri implant tissues.

## 1. Introduction

Alveolar ridge augmentation prior to dental implant placement is considered a safe and effective treatment option for the reconstruction of a deficient alveolar ridge [1]. Selecting the most suitable graft material for a patient relies on various considerations, including the anatomical structure, the shape and size of the bone defect, the type of prosthodontic rehabilitation, and the preferences of both the clinician and the patient [2]. While no studies conclusively demonstrate the superiority of any single bone augmentation technique, the surgeon should aim to choose a method that provides predictable results for the specific clinical scenario [3].

An ideal bone graft material should possess three key features: (a) osteoconduction, which provides a scaffold for new bone growth; (b) osteoinduction, which encourages the recruitment of cells that form bone and supports bone formation; and (c) osteogenesis, which involves the stimulation of cells within the graft to enhance bone regeneration [4]. Despite the development of various graft materials, autologous bone remains the gold standard for bone augmentation due to its possession of the three key properties mentioned [5]. The disadvantages of autologous bone substitutes are the need to open a donor surgical site and a high degree of resorption, up to 40% [6]. Other bone substitutes, including xenografts, allografts, and alloplastic materials, have been utilized over time but come with certain drawbacks [7,8,9]. Xenografts and alloplastic materials are limited to providing only osteoconduction, while allografts do not support osteoproliferation and pose a risk of disease transmission [4]. Given these facts, there is a necessity to develop an alternative graft material that will overcome these drawbacks.

Nampo et al. [10] investigated the potential for using teeth as a bone graft material for alveolar bone formation by comparing them to autogenous iliac bone grafts. They found that extracted teeth could be a promising option for bone grafts, as they demonstrate high predictability and reduced resorption post-grafting [10]. According to several experimental studies [11,12,13,14], extracted teeth demonstrate significant structural and biological potential to support the regeneration of bone defects. This information refers specifically to dentin, whose composition closely resembles bone, with a similar organic content (25% compared to 17.5%) and inorganic content (69.3% vs. 62%). The inorganic content of teeth includes four forms of calcium phosphate: hydroxyapatite, tricalcium phosphate, amorphous calcium phosphate, and octacalcium phosphate [15]. This inorganic material is recognized for its osteoconductive properties, making it a suitable option for bone augmentation. The organic component of dentin is primarily made up of a fibrous network of type I collagen, which accounts for 90% of its composition. The remaining 10% of the dentin matrix consists of non-collagenous proteins, such as osteocalcin, osteonectin, sialoprotein, and phosphoprotein, which play roles in bone calcification, along with growth factors like insulin-like growth factors, bone morphogenetic proteins (BMPs), and LIM mineralization protein 1. These components contribute to the osteoinductive properties of teeth [16]. Essentially, it was observed that dentin, whether used in particulate form or as a block graft, demonstrated both osteoconductive and osteoinductive characteristics.

A series of animal studies explored the effectiveness of autogenous tooth roots in a canine model for alveolar ridge augmentation and dental implant placement [17,18,19]. In these investigations, the roots, obtained from either healthy, non-infected, endodontically treated, or periodontally compromised teeth, were utilized as block grafts for bone augmentation of horizontal alveolar bone defects. Osteocalcin antigen reactivity and bone volume per tissue volume values were comparable between tooth root grafts and autologous bone block grafts [17]. Microcomputed tomography, immunohistochemistry, and histology analyses showed no significant differences between the autologous bone blocks taken from the retromolar region and the tooth grafts [18,19]. 

The outcomes of these animal studies were also confirmed by a series of human case reports by Schwarz et al. [20,21,22,23], claiming that autogenous tooth roots are comparable to conventionally used bone blocks in lateral alveolar ridge augmentation. In addition, there is no difference in the osseointegration of dental implants in alveolar ridges augmented with autogenous bone blocks or autogenous teeth. In a recent systematic review conducted by Guan et al. [24], it is concluded that the autogenous tooth bone graft is as effective as autogenous bone blocks in alveolar ridge reconstruction. They indicate that further research with a longer follow-up period is required to verify these conclusions. 

Taking into consideration these findings, the aim of this study was to evaluate the efficacy of alveolar ridge augmentation with autogenous tooth roots and staged implant placement, with an emphasis on peri-implant tissue stability in augmented sites. Additionally, this study aimed to assess the long-term clinical outcomes, including ridge width gain and peri-implant tissue health, to determine the viability of autogenous tooth roots as a grafting material. 

## 2. Materials and Methods

### 2.1. Study Design 

This case series study was conducted according to the guidelines of the Declaration of Helsinki and was approved by the Ethics Committee of the St. Catherine Specialty Hospital, Zagreb, Croatia (protocol code: 05-PA-30-XII-12/2019 on 5 December 2019) and registered via the Internet Portal of the Clinical Trials Register (NCT04678674). Prior to participation, every patient received a comprehensive explanation of the entire course of treatment and was required to sign written informed consent.

A total of 20 alveolar ridge augmentations in 15 patients with insufficient horizontal dimensions of mandibular alveolar ridge were performed to enable the placement of dental implants and subsequent fixed implant-prosthodontic rehabilitation. Mandibular or maxillary third molars without local pathological signs such as periapical lesions, pericoronitis or caries were used for alveolar ridge augmentation. In the absence of suitable wisdom teeth (third molars), periodontally compromised teeth were used. In total, 15 patients (8 males and 7 females; average age 40.6 years) with 20 horizontal ridge defects (cases) underwent augmentative surgery with autogenous tooth roots, of which 11 were augmented with impacted teeth and 9 with periodontally compromised teeth. Augmentation procedures were made from January till May 2019.

Sample size was calculated according to a series of similar case studies conducted by Schwarz et al. [20,21,22]. Accordingly, type I error was set to 0.05, and type II error was set to 0.20. Assuming a standard normal distribution for the power analysis, they calculated a minimum of 15 cases to achieve 95% power. Due to possible dropouts, 20 cases were included in this study.

### 2.2. Inclusion Criteria 

After a thorough explanation of the procedure and signing of the informed consent, patients were eligible for inclusion in the study if they met all of the following criteria: (a) individuals aged between 20 and 60 years, (b) presence of insufficient alveolar ridge width at the recipient site for implant placement (≤5.5 mm), (c) adequate bone height at the recipient site for implant placement (≥10 mm), and (d) keratinized tissue width ≥ 2 mm.

### 2.3. Exclusion Criteria

The study excluded patients who met any of the following criteria: (a) general contraindications for dentoalveolar surgical treatment; (b) uncontrolled diabetes mellitus (A1c test > 7%); (c) inflammatory or autoimmune disease present in the oral cavity; (d) prior treatment with bisphosphonates, immunosuppressants, antiresorptive, or high-dose corticosteroids (≥10 mg prednisolone (or equivalent) for >14 days); (e) malignant disease necessitating chemotherapy or radiotherapy within the last five years; (f) current smokers; and (g) pregnant or lactating women.

### 2.4. Therapeutic Outcomes—Alveolar Ridge Augmentation 

**Figure 1:**
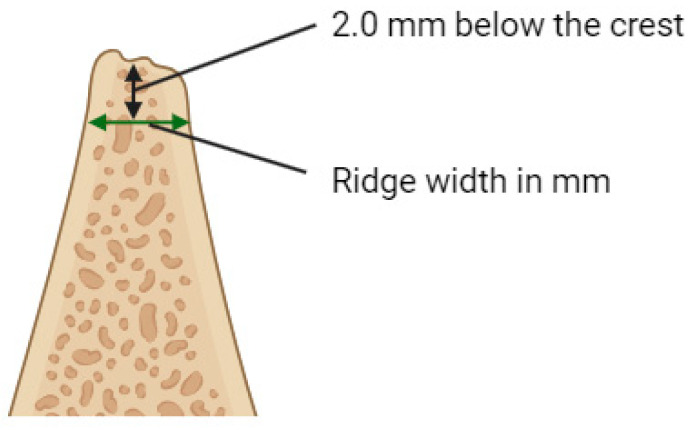
The measurement of alveolar ridge width. Created with BioRender.com.

The main outcome measure was the adequacy of the alveolar ridge width (RW), allowing the placement of dental implant of standard dimensions. Each patient underwent a cone-beam computed tomography (CBCT) scan on Dentsply Sirona Orthophos S 3D (Dentsply Sirona Inc., Charlotte, NC, USA) prior to surgery and 6 months post-surgery. RW was measured immediately before the augmentation procedure (RWb) and 6 months after surgery (RWa). Gain in ridge width (RWg [mm] = RWa − RWb) was calculated to evaluate the performance of the augmentative procedure. All measurements during radiographic evaluation were performed by one specialist in oral surgery at 2 mm below the crest of the alveolar ridge (Figure 1). 

### 2.5. Alveolar Ridge Augmentation Surgery

**Figure 2:**
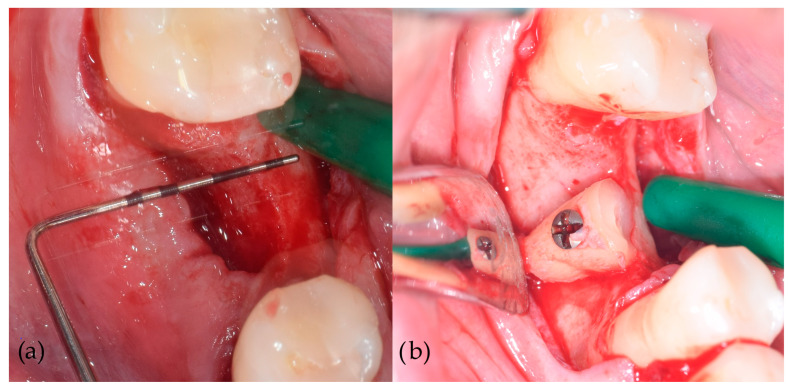
Alveolar ridge augmentation with autogenous tooth root. (**a**) Elevation of a full thickness mucoperiosteal flap, exposing the deficient alveolar ridge. (**b**) The fixation of the root graft to the recipient alveolar ridge.

After the administration of 4% articaine hydrochloride with adrenaline 1:200,000 (Ubistesin, 3M Deutschland GmbH, Neuss, Germany), partially/fully impacted wisdom teeth or periodontally compromised teeth were surgically removed. Following the removal of the respective teeth, the crowns were separated from the roots at the cemento- enamel junction using a sterile fissure carbide bur (H166A-021-HP, NTI-Kahla GmbH, Kahla, Germany) under sterile saline cooling. The separated tooth roots were reshaped with sterile round diamond bur (C081-035M-HP, NTI-Kahla GmbH, Kahla, Germany) according to the morphology of the bone defect. The superficial layer of cementum was removed with sterile flame diamond bur (864-016F-FG, NTI-Kahla GmbH, Kahla, Germany) until the underlying dentin was exposed to improve the tooth graft integration. Full thickness mucoperiosteal flaps were elevated using 15C surgical blade (HuFriedyGroup, Chicago, IL, USA) to expose the respective target sites. The recipient alveolar bone was flattened using a sterile round carbide bur (H141-027-HP, NTI-Kahla GmbH, Kahla, Germany) to ensure optimal tooth graft adherence to the recipient alveolar ridge, with additional drilling of decortication holes with smaller round carbide bur (H141-014-HP, NTI-Kahla GmbH, Kahla, Germany). The fixation of tooth root grafts was performed using one titanium osteosynthesis screw (1.5 × 9.5 mm) (Helmut Zepf Medizintechnik GmbH, Seitingen-Oberflacht, Germany) (Figure 2). After adequate fixation of the grafts, mucoperiosteal flaps were mobilized with periosteal releasing incisions to ensure tension-free closure and sutured in two layers with Resopren 5/0 (Resorba Medical GmbH, Nuremberg, Germany). 

Antibiotic therapy (Klavocin bid 875 mg amoxicillin + 125 mg clavulanic acid, Pliva, Zagreb, Croatia) was initiated twice daily for one day before surgery and then continued postoperatively for six days. Immediate postoperative care included intramuscular administration of 8 mg of dexamethasone, single dose. Non-steroidal anti-inflammatory drugs were prescribed for three postoperative days. Patients were directed to use a 0.12% chlorhexidine mouth rinse Curasept ADS 212 (Curaden AG, Kriens, Switzerland) twice a day for 10 days postoperatively. Suture removal was performed 10 days after surgery.

### 2.6. Implant Prosthodontic Rehabilitation 

**Figure 3:**
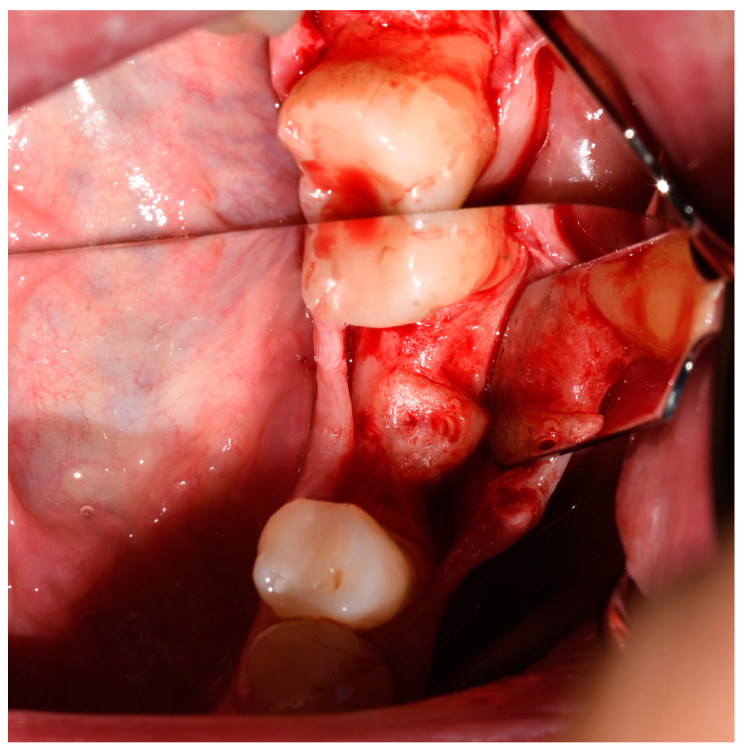
Homogenously integrated tooth root graft 6 months after alveolar ridge augmentation.

**Figure 4:**
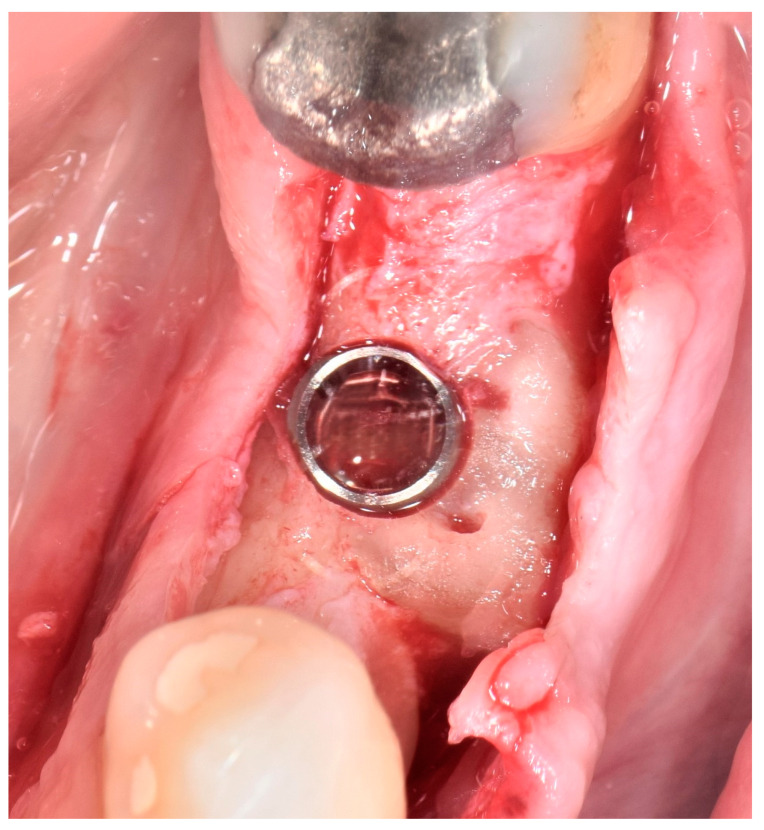
Epicrestally placed dental implant in the augmented site.

After a previously defined period of 6 months of healing, the patients underwent implant placement and subsequent prosthodontic rehabilitation. Full-thickness mucoperiosteal flaps were elevated under local infiltration anesthesia (4% articaine hydrochloride with adrenaline 1:200,000) to expose the augmented sites (Figure 3). Osteosynthesis screws were removed, and dental implants (Bone Level^®^ Tapered SLActive^®^, 4.1 × 10 mm RC, Institut Straumann AG, Basel, Switzerland) were placed epicrestally (Figure 4). Sixteen implants were placed in the mandibular first molar region, one implant was placed in mandibular second premolar region, and one implant was placed in mandibular canine region. Flaps were fixed with resorbable monofilament sutures Resopren 5/0 (Resorba Medical GmbH, Nuremberg, Germany). Sutures were removed 10 days postoperatively. 

Dental implants were restored with hybrid zirconia screw-retained crowns after a healing period of 10 weeks after placement. Zirconia crowns were cemented on Variobase^®^ abutments (Institut Straumann AG, Basel, Switzerland) using Multilink^®^ Hybrid Abutment cement (Ivoclar Vivadent, Schaan, Liechtenstein). The subgingival portion of the screw-retained zirconia crowns was polished and nonglazed. 

### 2.7. Therapeutic Outcomes—Clinical Measurements

Besides the abovementioned main radiographic outcome measurements regarding alveolar ridge augmentation performance, clinical measurements were performed at T1 (2 months after implant loading), T2 (3 years after implant loading), and T3 (5 years after implant loading). A plastic periodontal probe (UNC 12 Colorvue™ Probe, Hu-Friedy Group, Chicago, IL, USA) was used to conduct the following clinical tests: bleeding on probing (BOP), probing depth (PD), mucosal recession (MR), and clinical attachment level (CAL). 

BOP was evaluated as the absence or presence of bleeding within 30 s upon probing, PD was defined as the distance from the probe tip to the mucosal margin, MR was measured from the crown margin and the mucosal margin, and CAL was defined as the distance between the prosthodontic restoration margin and the bottom of the pocket (probe tip).

The clinical measurements were documented at six peri-implant positions, mesiobuccal, buccal, distobuccal, mesiolingual, lingual, and distolingual, by one specialist in periodontology. The measurements were assessed on two occasions, 24 h apart, to achieve strong reproducibility. The calibration was accepted in case of achieving ≤ 1 mm difference in 95% of the recordings.

### 2.8. Statistical Analysis 

The statistical analysis was performed using Microsoft Excel—Data analysis tools Version 2407 Build 16.0.17830.20166 (Microsoft Corporation, Redmond, WA, USA). Statistical analyses were performed using ANOVA test with a statistical significance level set to 0.05 with addition of descriptive statistics calculation. A paired t-test was used to compare the mean values of clinical measurements at different time points. Regarding the main outcome of alveolar ridge augmentation, appropriate descriptives, such as mean values, standard deviations, and medians, were calculated for alveolar ridge width. 

## 3. Results

### 3.1. Alveolar Ridge Augmentation Outcomes

**Figure 5:**
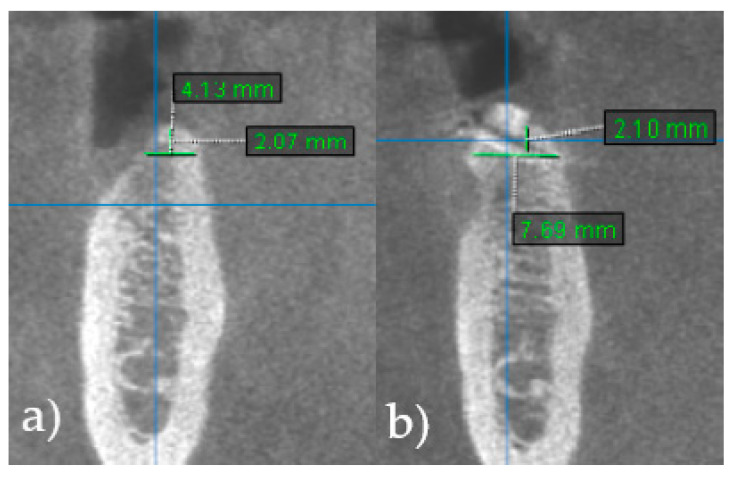
CBCT scans show horizontal dimension of the alveolar ridge. (**a**) Ridge width before (RWb) alveolar ridge augmentation surgery. (**b**) Ridge width 6 months after surgery (RWa), prior to implant placement.

Out of 20 cases, 18 resulted in successful augmentation, with the grafts integrating well with the alveolar bone and supporting subsequent implant placement (Figure 5), giving an overall success rate of 90% for the augmentative procedure performed in this study. However, complications were observed in two cases. One case experienced a wound dehiscence with subsequent infection during the healing period and was excluded from further investigation. Another case involved detachment of the autogenous tooth root graft from the host bone during the implant placement procedure and was also excluded from further analysis. 

Sixteen cases were performed in the mandibular molar region, one case in mandibular premolar region, and one in mandibular canine region. In 61% of the cases, the third molar was used as a donor tooth, while periodontally compromised teeth were used in the rest of the cases. 

Mean RWa values were 6.71 ± 0.74 mm, with mean RWg of 3.14 ± 0.54 mm. Descriptive parameters for alveolar ridge width values are presented in Table 1. 

### 3.2. Clinical Measurements 

A detailed overview of the BOP test is provided in Table 2. The data indicate that there was no significant increase in bleeding, as only one additional instance of evident bleeding was reported 3 and 5 years after implant loading. 

**Figure 6:**
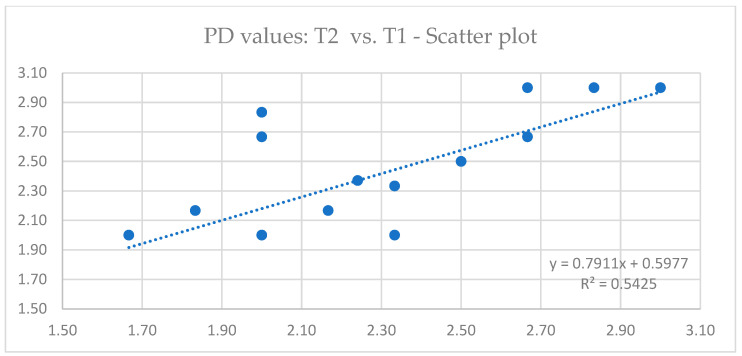
Linear regression scatter plot depicting the relationship between probing depth (PD) values 2 months (T1) and 3 years (T2) after implant loading.

**Figure 7:**
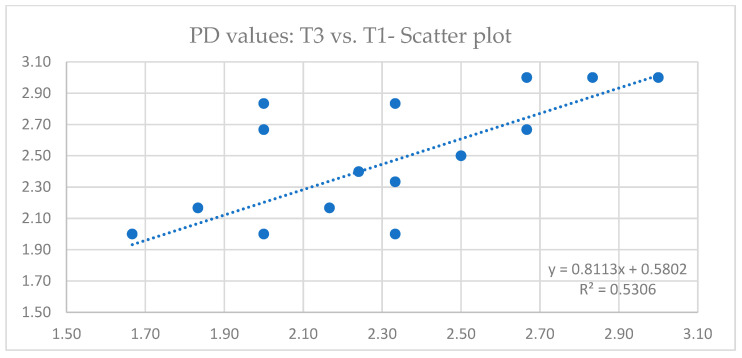
Linear regression scatter plot depicting the relationship between probing depth (PD) values 2 months (T1) and 5 years (T3) after implant loading.

Descriptive statistics of PD values are presented in Table 3. The data obtained by linear regression analysis revealed that there is a significant correlation between PD values at different time points (Figure 6 and Figure 7). Outputs of ANOVA tests are summarized in Table 4 (comparison T1 vs. T2) and Table 5 (comparison T1 vs. T3), where it can be observed that there was no statistically significant difference in PD values 3 years (*p* = 0.314) and 5 years (*p* = 0.233) after implant loading.

Given that mucosal recession (MR) was recorded in only two cases throughout the entire follow-up period, it was not feasible to conduct a separate statistical analysis of the data. However, the obtained data are provided in Table 6 for descriptive purposes. 

Finally, details of descriptive statistics for CAL values are summarized in Table 7. Outputs of ANOVA tests are presented in Table 8 (comparison T1 vs. T2) and Table 9 (comparison T1 vs. T3), with the conclusion that there is no statistically significant difference in CAL values among different follow-up periods (*p* = 0.220 for T2, and *p* = 0.123 for T3, respectively). 

## 4. Discussion

The presented study aimed to evaluate both the radiographic and long-term clinical outcomes of alveolar ridge augmentation with autogenous tooth roots and staged implant placement. Special emphasis was placed on clinical measurements, with important insights into the stability and health of peri-implant tissues over a period of up to five years post-implantation. Alveolar ridge augmentation using autogenous tooth roots enabled the successful placement of standard-sized dental implants, providing a stable environment for peri-implant tissues. The results of different clinical measurements indicate that dental implants can achieve and maintain long-term stability in terms of peri-implant tissue health. 

The mean ridge width before augmentation (RWb) was 3.14 ± 0.54 mm, while the mean ridge width after grafting (RWa) was 6.71 ± 0.74 mm. These measurements demonstrate the effectiveness of the augmentation procedure in increasing the ridge width to a level sufficient for implant placement. Conventional and commonly used techniques for the reconstruction of alveolar ridge often involve the use of particulate bone grafts, such as xenografts [25]. Even though these methods are thoroughly described in contemporary literature with well researched and predictable outcomes, there are certain disadvantages. While xenografts provide a scaffold for bone regeneration and have a predictable resorption rate, they possess only osteoconductive properties and sometimes require longer healing periods [26]. According to Janjua et al. [27], autogenous tooth grafts have proven to be useful in a multitude of clinical situations, particularly in the form of block grafts. Several clinicians have utilized block-type autogenous tooth for ridge augmentations [23,28,29], especially in cases where alveolar ridge defects measured 3 mm or more. Kim et al. [30] demonstrated in their long-term follow-up study that, in the majority of cases, implants placed in ridges augmented with autogenous tooth root grafts were ready for functional loading within 5-to-7 months. This firm graft integration with the recipient bone can be furtherly confirmed by a recent study conducted by Elraee et al. [31]. They have proven the existence of basal ankylosis and replacement resorption of the dentin matrix adherent to the alveolar bone. Furthermore, the histological analysis showed viable osteoprogenitor cells and new bone formation on the periphery of autogenous tooth root grafts. The high stiffness of exposed dentin prevents superficial resorption of the graft and supports the attachment of collagen fibers [32]. Besides the previously mentioned histological advantages, the application of mechanically stable block grafts additionally contributes to the efficacy of this type of bone-regeneration technique. Based on the findings of a recent systematic review by Guan et al. [24] comparing the efficacy of autogenous tooth blocks and autogenous bone blocks used for lateral ridge augmentation, the average increase in ridge width ranged from 3.52 ± 0.56 mm to 5.5 ± 1.73 mm in the autogenous tooth group. These values are comparable to the values demonstrated in the presented study. However, it is of vital importance to mention that a direct comparison of dimensional changes is by no means the only relevant criterion for treatment evaluation. Block grafts are intraoperatively shaped and adapted to the recipient bone defect; thus, it is up to the clinician to define the final shape and size of augmented site. Additionally, it was found that autogenous tooth root grafts had less average horizontal resorption than autogenous bone blocks [24]. A series of case studies by Schwarz et al. [18,20,21,22,23] have shown that bone augmentation with autogenous teeth can be an effective and highly predictable therapeutic approach. They claim that tooth root grafts have a comparable clinical outcome to autogenous bone blocks following lateral alveolar ridge augmentation and two-stage dental implant placement [22]. Moreover, they performed augmentation of fresh deficient extraction sockets, allowing for a subsequent placement of dental implants [21]. Korsch et al. [29] also concluded that tooth-shell technique has the comparable results to those of bone-shell technique regarding lateral ridge augmentation. It is worth mentioning that three cases in that study were affected by wound dehiscence, one grafted with autogenous tooth, and two with autogenous bone block. In the presented study, there was also one complication of wound dehiscence following the infection of the graft. According to the findings of the contemporary literature [24], the most reported postoperative complications associated with autogenous tooth root grafts include wound dehiscence, the fixation screw exposure, and peri-implant mucositis. Nevertheless, these events are not correlated with notable percentage of alveolar ridge augmentation failure [33]. Less frequent postoperative complications, a lower resorption rate, and the absence of the donor site morbidity are substantial factors indicating the superior reliability of tooth root grafts compared to alveolar bone blocks. Still, the lack of long-term follow-up studies hinders the ability to draw a definitive conclusion [34]. 

Clinical measurements gave valuable insights into long-term peri-implant tissue stability over a period of up to five years. No statistically significant differences were observed for clinical parameters (BOP, PD, MR, and CAL) among different follow-up visits. These results are in accordance with a prospective controlled clinical study conducted by Schwarz et al. [22]. They have not found statistically significant differences in mean PD, BOP, MR, and CAL values after a follow-up period of 44 weeks. The short-term follow-up outcomes of their study are comparable to long-term (>3 years) outcomes of lateral bone augmentation procedures, revealing no significant changes in BOP values over time [35]. The important observation of relatively high BOP values in the previously mentioned study [22] should be addressed and compared with those in the presented study. They recorded baseline clinical measurements immediately after insertion of the fixed prosthodontic restorations at respective implant sites. Consequently, BOP values were increased as the result of trauma induced by insertion of the prosthodontic restorations. The completion of peri-implant soft-tissue healing was taken into consideration, and the first follow-up visit with clinical measurements was two months after implant loading in the current study. Furthermore, there was no significant difference in PD values over a follow-up period of five years. The mean PD was 2.24 ± 0.37 mm two months after implant loading, and it was 2.40 ± 0.41 after five years. These values are comparable to those of a prospective study by Pohl et al. [36], where peri-implant PD was 1 mm after one year and 2 mm after two years. MR was recorded in only two cases throughout the entire follow-up period, which is in accordance with the absence of any MR in study by Schwarz et al. [22].

In summary, this study does not lack some limitations. One limitation of this study was the absence of histological analysis. Ethical considerations prevented the collection of core biopsies for additional histological analysis in this study. However, previous research has shown that autogenous dentin can integrate with the host bone, resulting in new bone formation [37,38]. Peri-implant tissue stability confirmed with clinical measurements and the radiological findings of this study support these observations. Additionally, another limitation was that the CBCT scans were only taken 6 months after augmentation, prior to implant placement, thus limiting the evaluation of the extent of augmentation resorption during follow-up period. 

While these results are encouraging, it is important to highlight that this is one of the few clinical studies focusing on the efficacy and stability of augmented tooth roots with long-term follow-up. Therefore, the impact on implant survival and success must be thoroughly assessed in future research. 

## 5. Conclusions

The findings of this study highlight the potential of autogenous tooth root grafts for alveolar ridge augmentation. According to the presented results, this approach allows for the successful placement of standard-sized dental implants and provides a stable environment for peri-implant tissues over a long-term period. Considering the discussed limitations, autogenous tooth roots could serve as a substitute for the different modalities of alveolar ridge augmentation in everyday clinical practice. 

## Figures and Tables

**Table 1:** Alveolar ridge augmentation outcomes with values of ridge width before augmentation (RWb), ridge width after augmentation (RWa), and gain in ridge width (RWg) (*n* = 18 cases).

	RWb (mm)	RWa (mm)	RWg (mm)
Mean	3.52	6.71	3.14
SD	0.58	0.74	0.54
Median	3.51	6.52	3.03

**Table 2:** Bleeding on probing (BOP) results for different time points.

BOP	T1 (2 Months)	T2 (3 Years)	T3 (5 Years)
Present	3	4	4
Absent (not present 30 s after probing)	15	14	14
Total	18	18	18
% of present bleeding	16.7	22.2	22.2

**Table 3:** Descriptive statistics for probing depth (PD).

	T1 (2 Months)	T2 (3 Years)	T3 (5 Years)
Mean	2.24	2.37	2.40
SD	0.37	0.39	0.41
Median	2.08	2.25	2.25
Min	1.67	2.00	2.00
Max	3.00	3.00	3.00

**Table 4:** Summary of ANOVA test (PD) for T1 (2 months) and T2 (3 years) after implant loading.

Source of Variation	SS	df	MS	F	*p*-Value	F Crit
Between groups	0.1512	1	0.1512	1.0426	0.3144	4.1300
Within groups	4.9321	34	0.1451			
Total	5.0833	35				

**Table 5:** Summary of ANOVA test (PD) for T1 (2 months) and T3 (5 years) after implant loading.

Source of Variation	SS	df	MS	F	*p*-Value	F Crit
Between groups	0.2230	1	0.2230	1.4776	0.2325	4.1300
Within groups	5.1312	34	0.1509			
Total	5.3542	35				

**Table 6:** Mucosal recession (MR) results for different time periods.

	T1 (2 Months)	T2 (3 Years)	T3 (5 Years)
Mean	0.00	0.11	0.11
SD	0.00	0.37	0.37
Median	0.00	0.00	0.00
Min	0.00	0.00	0.00
Max	0.00	1.50	1.50

**Table 7:** Descriptive statistics for clinical attachment level (CAL).

	T1 (2 Months)	T2 (3 Years)	T3 (5 Years)
Mean	2.24	2.45	2.51
SD	0.37	0.63	0.62
Median	2.09	2.25	2.42
Min	1.67	2.00	2.00
Max	3.00	4.50	4.50

**Table 8:** Summary of ANOVA test (CAL) for T1 (2 months) and T2 (3 years) after implant loading.

Source of Variation	SS	df	MS	F	*p*-Value	F Crit
Between groups	0.4103	1	0.4103	1.5596	0.2203	4.1300
Within groups	8.9450	34	0.2631			
Total	9.3554	35				

**Table 9:** Summary of ANOVA test (CAL) for T1 (2 months) and T3 (5 years) after implant loading.

Source of Variation	SS	df	MS	F	*p*-Value	F Crit
Between groups	0.6498	1	0.6498	2.5065	0.1226	4.1300
Within groups	8.8147	34	0.2593			
Total	9.4645	35				

## Data Availability

The data presented in this study are available upon request from the corresponding author.
